# Variations in disability and quality of life with age and sex between eight lower income and middle-income countries: data from the INDEPTH WHO-SAGE collaboration

**DOI:** 10.1136/bmjgh-2017-000508

**Published:** 2017-12-20

**Authors:** Francesc Xavier Gomez-Olive, Julia Schröders, Isabella Aboderin, Peter Byass, Somnath Chatterji, Justine I Davies, Cornelius Debpuur, Siddhivinayak Hirve, Abraham Hodgson, Sanjay Juvekar, Kathleen Kahn, Paul Kowal, Rose Nathan, Nawi Ng, Abdur Razzaque, Osman Sankoh, Peter K Streatfield, Stephen M Tollman, Siswanto A Wilopo, Miles D Witham

**Affiliations:** 1 MRC/Wits Rural Public Health and Health Transitions Research Unit, School of Public Health, Faculty of Health Sciences, University of the Witwatersrand, Johannesburg, South Africa; 2 Department of Public Health and Clinical Medicine, Epidemiology and Global Health Unit, Umeå University, Umeå, Sweden; 3 African Population and Health Research Center, Nairobi, Kenya; 4 Centre for Research on Ageing, University of Southampton, Southampton, UK; 5 OPTENTIA Research Focus, North West University, Vanderbijlpark, South Africa; 6 Department of Health Statistics and Informatics, World Health Organization, Geneva, Switzerland; 7 Institute for Global Health, King’s College London, London, UK; 8 Navrongo Health Research Centre, Navrongo, Ghana; 9 Vadu Rural Health Program, KEM Hospital Research Centre, Pune, India; 10 INDEPTH Network, Accra, Ghana; 11 Ifakara Health Institute, Dar es Salaam, Tanzania; 12 Matlab HDSS, Matlab, Bangladesh; 13 Department of Mathematics and Statistics, Njala University, Njala, Sierra Leone; 14 School of Public Health, University of the Witwatersrand, Johannesburg, South Africa; 15 HDSS Purworejo and Faculty of Medicine, Universitas Gadjah Mada, Yogyakarta, Indonesia; 16 Department of Ageing and Health, School of Medicine, University of Dundee, Dundee, UK

**Keywords:** other infection, disease, disorder, or injury, cross-sectional survey, epidemiology

## Abstract

**Background:**

Disability and quality of life are key outcomes for older people. Little is known about how these measures vary with age and gender across lower income and middle-income countries; such information is necessary to tailor health and social care policy to promote healthy ageing and minimise disability.

**Methods:**

We analysed data from participants aged 50 years and over from health and demographic surveillance system sites of the International Network for the Demographic Evaluation of Populations and their Health Network in Ghana, Kenya, Tanzania, South Africa, Vietnam, India, Indonesia and Bangladesh, using an abbreviated version of the WHO Study on global AGEing survey instrument. We used the eight-item WHO Quality of Life (WHOQoL) tool to measure quality of life and theWHO Disability Assessment Schedule, version 2 (WHODAS-II) tool to measure disability. We collected selected health status measures via the survey instrument and collected demographic and socioeconomic data from linked surveillance site information. We performed regression analyses to quantify differences between countries in the relationship between age, gender and both quality of life and disability, and we used anchoring vignettes to account for differences in interpretation of disability severity.

**Results:**

We included 43 935 individuals in the analysis. Mean age was 63.7 years (SD 9.7) and 24 434 (55.6%) were women. In unadjusted analyses across all countries, WHOQoL scores worsened by 0.13 points (95% CI 0.12 to 0.14) per year increase in age and WHODAS scores worsened by 0.60 points (95% CI 0.57 to 0.64). WHODAS-II and WHOQoL scores varied markedly between countries, as did the gradient of scores with increasing age. In regression analyses, differences were not fully explained by age, socioeconomic status, marital status, education or health factors. Differences in disability scores between countries were not explained by differences in anchoring vignette responses.

**Conclusions:**

The relationship between age, sex and both disability and quality of life varies between countries. The findings may guide tailoring of interventions to individual country needs, although these associations require further study.

**Key questions**

**What is already known about this topic?**

- Disability and quality of life are key outcomes in promoting healthy ageing, and worse quality of life and disability are both associated with higher mortality.
- Little is known about their determinants in lower income and middle-income countries.

**What are the new findings?**

- Marked differences exist between the analysed sites in how both disability and quality of life change with age, with significant differences between sexes.
- The underlying causes of these differences are not clear.

**Recommendations for policy**

- By increasing awareness of the importance of quality of life and disability in low-income and middle-income countries.
- Thus aiding countries in their efforts to promote healthy ageing and minimise the progression of disability as populations age.

## Introduction

Accelerated population ageing is now well established in many lower and middle-income countries (LMICs), leading to dramatic increases in both the absolute number of older people and the proportion of older people in LMICs.^1^ Data from high-income countries (HICs) show worsening disability with age^2 3^ and differences in disability-free life expectancy between men and women.^4^ In HICs, disability and quality of life are the outcomes that are most important to older people themselves.^5^ Maintenance of ability and good quality of life are also important goals for government and society, as intrinsically worthwhile to citizens and also because self-reported disability and quality of life both strongly predict risk of death^6 7^ and because of the need to mitigate spiralling health and social care costs for those with disability.^8^ As LMICs continue to transition socioeconomically and demographically, maintenance of ability and quality of life will likely become a key focus for governments working towards developing responsive health and long-term care systems.^9^

Although some data are available on disability and quality of life, and their determinants, within individual LMICs,^10–18^ very little work has been done to explore if these metrics are experienced by ageing populations in different countries in the same way. Such knowledge would inform debates on potential cross-cutting approaches to the development of health and long-term care systems and on key individual and structural-level factors (eg, variations in comorbid disease patterns and socioeconomic status) driving differential experiences of health and well-being in old age across societies. Understanding between-country differences would provide valuable insights into how countries might shape economic, health and social policies to improve disability and quality of life.

The aim of this paper is therefore to describe how self-reported disability and self-reported quality of life vary with age and sex across eight LMICs in Africa and Asia and to explore what other factors might explain differences in disability and quality of life using data from the International Network for the Demographic Evaluation of Populations and their Health (INDEPTH) and WHO Study on global AGEing (WHO-SAGE) dataset.

## Methods

### Populations and dataset

This analysis used data from the multicentre INDEPTH WHO-SAGE study, conducted in 2006–2007 across eight health and demographic surveillance system (HDSS) sites in LMICs in Africa (Ghana, Kenya, Tanzania and South Africa) and Asia (Bangladesh, Indonesia, India and Vietnam) using a short version of the national WHO-SAGE questionnaire. The methods for the study have been described in detail previously,^19^ but in brief, individuals aged 50 years and over from eight HDSS sites of the INDEPTH (www.indepth-network.org) were selected for interview. Participants had to be registered on the HDSS to be invited and had to be able to give informed consent to participate. Participants with dementia or difficulty communicating were not excluded on the basis of these impairments unless they precluded giving consent or completing the questionnaires. A total of 58 004 individuals were invited to participate; 46 269 agreed to take part. All 2334 participants with incomplete demographic or socioeconomic data were excluded from the final dataset as previously described^20^; complete data were available for 43 935 individuals who form the dataset analysed in this study.

### Variables

The survey used the abbreviated INDEPTH WHO-SAGE survey instrument, which has been described previously.^20^ We measured disability using the validated WHO Disability Assessment Schedule, version 2 (WHODAS-II) disability score.^21^ This score measures impairments in selected basic and instrumental activities of daily living across six domains (mobility, self-care, cognition, interaction with others, life activities and social participation); measures are summed to form a composite score, then normalised to a 0–100 scale, where 0 represents no disability and 100 represents the worst disability. We measured quality of life using the validated eight-item EUROHIS-WHOQoL score,^22^ an abbreviated version of the 26-item WHOQoL-BREF score.^23^ Each item is scored on a five-point scale and summed; for this analysis, we then normalised the score to a 0–100 scale, with 100 denoting the best quality of life. Previous studies have shown good agreement between the EUROHIS-WHOQoL and the WHOQoL-BREF across a number of countries.^24^

To account for differences in interpretation and perception of disability severity between individuals (which may vary between countries, gender, age or other factors), one in four participants in six of the participating sites (all except Ghana and South Africa) answered anchoring vignette questions. As described previously,^19^ these vignettes allowed individuals to score a concrete example of disability on the same scales as used in WHODAS, allowing the WHODAS responses for each individual to be calibrated against standard scenarios of varying severity. The aim of this approach was to account for differences in how a given level of disability (and hence potentially their own level of disability) would be perceived and scored by different individuals. Each vignette posed a scenario based on a daily activity, and then asked the respondents how much difficulty the person in the vignette had with each activity on a five-point scale (1=no problem; 5=extreme problem or unable to perform activity). Previous work on related vignettes (covering mobility and cognition) suggests that although the psychometric performance of vignettes in WHO-SAGE is imperfect at predicting anchoring in absolute terms, the vignettes still provide information on how individuals order severity of conditions, particularly for self-care.^25^ The vignettes covered: a person able to wash, bathe and dress himself slowly; a person housebound because of arthritis who cannot dress and needs help with washing; a person able to self-care except for occasional help with bathing and dressing when he has back pain; and a person requiring help with all care because of paralysis from the neck down. For each vignette, we asked a question on ability to self-care and a question on maintaining appearance, corresponding to questions asked on self-care and appearance within WHODAS-II. These data were not available for Ghana or South Africa. To determine whether differences in perception of impairment affected how disability scores varied between countries, we used data from these four vignettes (eight questions) in multivariable analyses.

We determined family size as the total number of people in the household at the most recent HDSS survey round. We derived a measure of socioeconomic status as quintiles of a wealth index as previously described.^20^ We dichotomised living status as living alone or living with others; we dichotomised marital status as either currently in a partnership or not (encompassing all of never married, widowed and divorced/separated). We categorised years of education as no formal education, primary or less than 6 years of formal education and six or more years of formal education; this categorisation reflected the questions on education asked at each site. We took responses for selected markers of health status from five-point scales (1=no problem; 5=extreme problem or unable to do) for problems in the last 30 days with: feeling sad, low or depressed; problems with worry or anxiety; problems with bodily aches or pain; problems with bodily discomfort; difficulties concentrating or remembering things; problems in learning a new task; problems seeing a person or object at 20 m (far vision); and problems seeing an object at arms’ length (near vision).

### Analyses

We generated descriptive statistics by age and sex category for WHODAS-II and WHO Quality of Life (WHOQoL) score (using age categories in 5-year bands from 50 years to 54 years, from 75 years to 79 years, then 80 years and over). We used medians for WHODAS-II data as these were not normally distributed. We plotted scores against age category for each country, with men and women plotted separately.

To examine associations of potential explanatory variables with quality of life, we undertook linear regression modelling with forced entry of variables using WHOQoL as the dependent variable. Both health status and disability would be expected to be associated with quality of life. However, because health status markers and WHODAS-II scores were highly correlated,^20^ we chose not to enter both into the same model. Instead, we ran three models: one with WHODAS-II as an independent variable, another with answers to individual health status questions and a third using a summary health status score, previously derived and weighted using Item Response Theory.^20^ For analysis of potential explanatory variables with disability, we used WHODAS-II as the dependent variable. To account for the non-normal distribution in analysis of WHODAS-II, we used generalised linear modelling with a Tweedie probability distribution (power 1.9) and identity link. This distribution was selected due to the large number of individuals with a score of zero, plus significant skewing of the non-zero values. A simple linear regression could therefore not be employed even after transforming the WHODAS-II scores, and we therefore selected this Tweedie distribution as the closest approximation to the observed distribution of the WHODAS-II scores. All regression models included population weighting factors for age and sex and included terms to explore the interaction between age and country, sex and country, and age, sex and country with the dependent variable. We used South Africa as the referent country in each analysis comparing other countries with South Africa. South Africa was the country with the lowest life expectancy and highest mortality in 2006/2007 yet had the highest levels of gross domestic product per capita, urbanisation and industrialisation.^26^ As such, it provides a comparator at one end of multiple distributions, particularly those involving development and industrialisation that other countries are moving towards. We decided the choice of explanatory variables for both models a priori; we included factors likely to be on a causal pathway or representing unmeasured constructs on a causal pathway (eg, age as a proxy for disease and frailty; family size and marital status as possible proxies for social support). For both sets of models, variables included age, sex, country, socioeconomic status, marital status, living arrangements and family size. Data on comorbid disease were not available in the INDEPTH WHO-SAGE dataset. The health status markers included in the WHOQoL analyses were anxiety and depression, and selected measures of health including vision problems, pain and discomfort and problems concentrating and learning a new task. We included education in the WHOQoL analysis given previous work linking education level to life satisfaction.

We constructed a separate regression model for the dataset (n=8201) where vignette data on self-care were available. This analysis examined determinants of disability as above, with and without the vignette results, to test whether inclusion of the vignette data changed the association between baseline variables and WHODAS-II scores. We calculated the difference for each individual between the vignette question score and the score on the corresponding WHODAS disability domain; we then included this difference for each of the eight vignette questions in the model as a covariate in multivariable analyses; scores from different vignette questions were not combined prior to use in the regression analysis. We used generalised linear modelling using a Tweedie distribution as above with log-link for this analysis. We undertook all analyses using SPSS V.24; we took a two-sided P value of <0.05 as statistically significant for all analyses.

## Results

**Figure 1:**
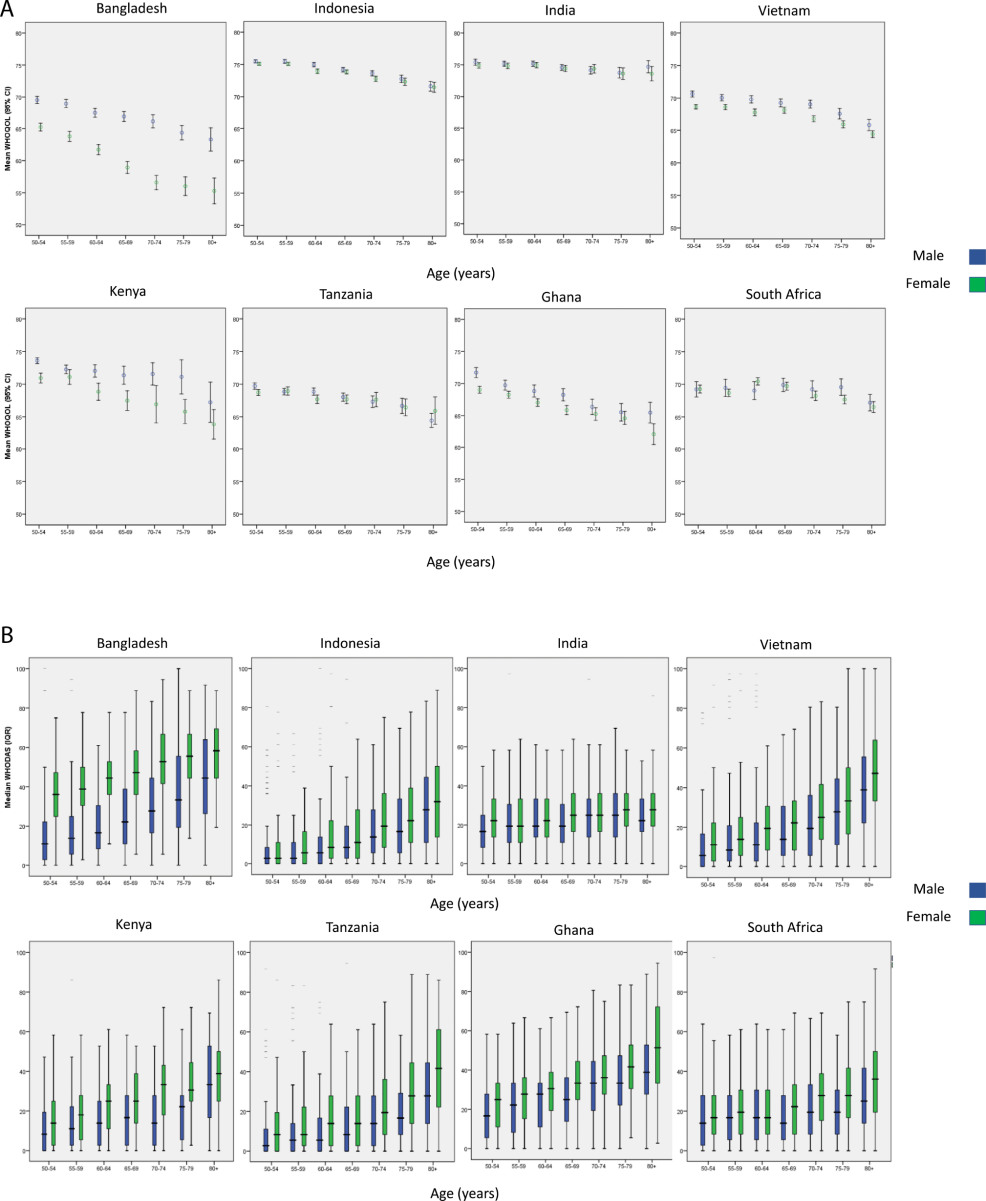
(A) Mean WHOQoL scores by age category, country and sex. Error bars represent 95% CI. WHOQoL transformed to 0–100 scale (100=best quality of life). (B) Median WHODAS scores by age category, country and sex. Boxes represent interquartile range. WHODAS score transformed to 0–100 scale (100=worst disability). WHODAS, WHO Disability Assessment Schedule; WHOQoL, WHO Quality of Life.

We included data from a total of 43 935 people in the current analysis (table 1). Figure 1 shows the relationships, separately for men and women, between age and quality of life (figure 1A) and between age and disability (figure 1B), across the eight included sites. Unadjusted analysis showed a mean worsening in WHOQoL score across all sites of 0.13 points (95% CI 0.12 to 0.14) for every 1 year increase in age. For WHODAS-II, scores worsened by 0.60 points (95% CI 0.57 to 0.64) for every year increase in age. These mean results mask substantial variability in the relationship between covariates and these outcomes between countries, as shown by the results of within-country regression analyses in online supplementary tables 1 and 2. Most sites showed higher disability levels among women than among men at ages 50–54 years, with an even greater disparity between the sexes at age 80+ years. The exception was the Bangladesh site, where a very large sex difference in disability at ages 50–54 years was somewhat narrower at age 80+ years. The spread of disability scores across sites was much greater at age 50–54 years for women than for men and remained greater across the age range;, but for women, disability scores converged between sites in the oldest age categories. For quality of life, most sites showed similar scores for men and women at all ages. The exception, again, was the Bangladesh site, where quality of life was worse for women across ages, particularly so among the oldest-old. Scores for both sexes showed some decline and greater cross-site heterogeneity with age. The India and Indonesia sites, which maintained the highest scores of all sites at all ages, showed a less marked decline in scores with age.

10.1136/bmjgh-2017-000508.supp1 Supplementary file 1

**Table 1:** Baseline details of included populations

	Bangladesh	Ghana	Indonesia	India	Kenya	Tanzania	Vietnam	South Africa	All
N	4004	4294	11 753	4514	1991	5024	8516	3839	43 935
Mean age (years) (SD)	62.1 (9.0)	62.6 (9.0)	64.1 (9.4)	62.9 (8.9)	59.0 (8.9)	62.5 (9.2)	65.3 (10.7)	66.5 (10.6)	63.7 (9.7)
50–54 years (%)	1008 (25.2)	918 (21.4)	2415 (20.5)	800 (17.7)	830 (41.7)	1139 (22.7)	1721 (20.2)	608 (15.8)	9439 (21.5)
55–59 years (%)	804 (20.1)	909 (21.2)	1929 (16.4)	968 (21.4)	488 (24.5)	1052 (20.9)	1492 (17.5)	607 (15.8)	8249 (18.8)
60–64 years (%)	762 (19.0)	851 (19.8)	1875 (16.0)	856 (19.0)	290 (14.6)	907 (18.1)	1127 (13.2)	546 (14.2)	7214 (16.4)
65–69 years (%)	591 (14.8)	701 (16.3)	2170 (18.5)	835 (18.5)	149 (7.5)	749 (14.9)	1126 (13.2)	598 (15.6)	5919 (13.5)
70–74 years (%)	414 (10.3)	390 (9.1)	1637 (13.9)	540 (12.0)	81 (4.1)	572 (11.4)	1152 (13.5)	509 (13.3)	5295 (12.1)
75–79 years (%)	273 (6.8)	333 (7.8)	1007 (8.6)	288 (6.4)	67 (3.4)	338 (6.7)	930 (10.9)	520 (13.5)	3756 (8.5)
80+ years (%)	152 (3.8)	192 (4.5)	720 (6.1)	227 (5.0)	86 (4.3)	267 (5.3)	968 (11.4)	451 (11.7)	3063 (7.0)
Female sex (%)	2005 (50.1)	2660 (61.9)	6333 (53.9)	2163 (47.9)	693 (34.8)	2636 (52.5)	5054 (59.3)	2890 (75.3)	24 434 (55.6)
Mean WHOQoL score (SD)	22.6 (5.6)	20.7 (4.8)	16.7 (3.4)	16.2 (3.0)	18.4 (4.2)	20.8 (3.6)	20.4 (4.3)	20.0 (4.4)	19.1 (4.6)
Median WHODAS score (IQR)	33.3 (16.7–49.3)	27.8 (16.7–38.9)	8.3 (2.8–22.2)	22.2 (13.9–33.3)	13.9 (2.8–27.8)	11.1 (2.8–25.0)	16.7 (5.6–36.1)	22.2 (8.3–33.3)	16.7 (5.6–33.3)
No formal education (%)	2257 (56.4)	0 (0)	3440 (29.3)	259 (5.7)	566 (28.4)	1983 (39.5)	877 (10.3)	2510 (65.4)	11 892 (27.1)
Primary or <6 years (%)	1149 (28.7)	3980 (92.7)	6459 (55.0)	3221 (71.4)	1125 (56.5)	2836 (56.4)	4182 (49.1)	746 (19.4)	23 698 (53.9)
6 years or more (%)	598 (14.9)	314 (7.3)	1854 (15.8)	1034 (22.9)	300 (15.1)	205 (4.1)	3457 (40.6)	583 (15.2)	8345 (19.0)
Married or cohabiting (%)	3049 (76.1)	2300 (53.6)	8400 (71.5)	3595 (79.6)	1369 (68.8)	3351 (66.7)	5882 (69.1)	1775 (46.2)	29 721 (67.6)
Living alone (%)	112 (2.8)	199 (4.6)	908 (7.7)	99 (2.2)	479 (24.1)	109 (2.2)	565 (6.6)	219 (5.7)	2690 (6.1)
Quintile of socioeconomic status (%)									
(Poorest) 1	611 (15.3)	1210 (28.2)	2394 (20.4)	514 (11.4)	483 (24.3)	950 (18.9)	1209 (14.2)	616 (16.0)	7987 (18.2)
2	667 (16.7)	1064 (24.8)	2317 (19.7)	686 (15.2)	302 (15.2)	982 (19.5)	1548 (18.2)	728 (19.0)	8294 (18.9)
3	701 (17.5)	958 (22.3)	2390 (20.3)	994 (22.0)	406 (20.4)	1056 (21.0)	1787 (21.0)	739 (19.2)	9031 (20.6)
4	930 (23.2)	808 (18.8)	2387 (20.3)	963 (21.3)	422 (21.2)	2036 (40.5)	1996 (23.4)	812 (21.2)	10 354 (23.6)
(Richest) 5	1095 (27.3)	254 (5.9)	2265 (19.3)	1357 (30.1)	378 (19.0)	0 (0)	1976 (23.2)	944 (24.6)	8269 (18.8)
Mean family size (SD)	5.4 (2.5)	6.6 (3.9)	3.5 (1.7)	6.9 (3.5)	4.1 (3.2)	10.2 (5.7)	4.2 (2.0)	7.1 (4.0)	5.6 (3.9)
On a scale of 1 best/no problem to 5=worst/cannot do or severe problem									
Median depressive symptoms (IQR)	3 (2–4)	2 (1–3)	1 (1–2)	2 (1–2)	2 (1–2)	1 (1–2)	1 (1–2)	2 (1–3)	2 (1–2)
Median anxiety symptoms (IQR)	4 (2–4)	2 (2–3)	1 (1–2)	2 (1–3)	1 (1–2)	1 (1–2)	1 (1–3)	3 (1–4)	2 (1–3)
Median bodily pain (IQR)	3 (2–4)	2 (2–3)	2 (1–3)	2 (1–3)	2 (1–3)	2 (1–3)	2 (2–4)	3 (1–3)	2 (1–3)
Median discomfort (IQR)	3 (2–4)	2 (2–3)	2 (1–3)	2 (1–3)	2 (1–3)	2 (1–3)	2 (1–4)	3 (1–3)	2 (1–3)
Median problems concentrating (IQR)	3 (1–4)	2 (1–3)	2 (1–2)	2 (1–2)	1 (1–3)	1 (1–2)	2 (1–3)	2 (1–3)	2 (1–3)
Median problems task learning (IQR)	2 (1–3)	2 (2–3)	2 (1–3)	2 (1–2)	1 (1–2)	1 (1–2)	2 (1–3)	2 (1–3)	2 (1–3)
Median problems far vision (IQR)	2 (1–4)	2 (1–3)	2 (1–3)	2 (1–3)	1 (1–2)	1 (1–3)	1 (1–3)	1 (1–2)	2 (1–3)
Median problems near vision (IQR)	2 (1–3)	1 (1–2)	2 (1–3)	2 (1–3)	2 (1–3)	1 (1–2)	1 (1–2)	1 (1–2)	1 (1–2)

WHODAS, WHO Disability Assessment Schedule; WHOQoL, WHO Quality of Life.

Tables 2 and 3 show the results of the regression models, showing which variables are associated with quality of life and disability levels and highlighting the variability between countries in how quality of life and disability change with age. For quality of life, disability, marital status, education, living arrangements, family size and socioeconomic variables were significantly associated with quality of life in adjusted models; in models where health status was substituted for WHODAS score (online supplementary tables 3 and 4), health status was significantly associated with quality of life. The gradient of WHOQOL score with advancing age was significantly different between South Africa (the referent country) and Bangladesh, Ghana, Kenya and Tanzania, as shown by the significant interaction terms. Similarly, the relationship between sex and WHOQOL score was significantly different for Bangladesh, Tanzania and Vietnam when compared with South Africa as referent. Finally, the magnitude of difference in the age-WHOQOL gradient between men and women was significantly different to South Africa for Bangladesh, Indonesia, Tanzania and Vietnam, as shown by the significant age*sex*country interaction terms.

**Table 2:** Regression model for associations with self-reported quality of life (WHOQoL)

Variable	B (SE)	P
Age (per year)	0.026 (0.017)	0.12
Country of residence		
Bangladesh	2.324 (1.373)	0.09
Ghana	7.988 (1.413)	<0.001
Indonesia	3.496 (1.198)	0.004
India	6.914 (1.350)	<0.001
Kenya	6.454 (1.607)	<0.001
Tanzania	2.401 (1.347)	0.08
Vietnam	−2.131 (1.258)	0.09
South Africa	Referent	–
Female sex	−0.010 (1.259)	0.99
Education		
None	−0.901 (0.098)	<0.001
<6 years	−0.615 (0.080)	<0.001
≥6 years	Referent	–
Marital status	−0.862 (0.069)	<0.001
Living arrangements	0.222 (0.123)	0.07
Socieconomic status		
1 (lowest)	Referent	–
2	0.507 (0.087)	<0.001
3	1.074 (0.086)	<0.001
4	1.328 (0.084)	<0.001
5 (highest)	2.405 (0.092)	<0.001
Family size	−0.032 (0.009)	<0.001
WHODAS	−0.180 (0.002)	<0.001

Age*sex*country interactions						
	Age*country		Sex*country		Age*sex*country	
	B (SE)	P	B (SE)	P	B (SE)	P
Bangladesh	−0.057 (0.021)	0.007	9.742 (1.748)	<0.001	−0.186 (0.019)	<0.001
Ghana	−0.109 (0.022)	<0.001	−2.731 (1.731)	0.12	0.034 (0.019)	0.07
Indonesia	0.004 (0.019)	0.83	−1.508 (1.431)	0.29	0.032 (0.011)	0.003
India	−0.021 (0.021)	0.31	0.014 (1.705)	0.99	0.011 (0.019)	0.55
Kenya	−0.071 (0.026)	0.007	0.250 (2.188)	0.91	−0.018 (0.030)	0.54
Tanzania	−0.062 (0.021)	0.003	−5.638 (1.639)	0.001	0.103 (0.017)	<0.001
Vietnam	0.024 (0.019)	0.23	−2.978 (1.476)	0.04	0.041 (0.012)	0.001
South Africa	Referent	–	Referent	–	0.013 (0.019)	0.49

B, unstandardised coefficient; WHODAS, WHO Disability Assessment Schedule; WHOQoL, WHO Quality of Life.

**Table 3:** Regression model for associations with self-reported disability (WHODAS)

Variable	B (SE)	P
Age (per year)	0.218 (0.102)	0.03
Country of residence		
Bangladesh	−32.589 (8.235)	<0.001
Ghana	−21.923 (9.519)	0.02
Indonesia	−27.450 (6.549)	<0.001
India	2.295 (8.303)	0.78
Kenya	−18.299 (8.851)	0.04
Tanzania	−22.579 (7.022)	0.001
Vietnam	−30.730 (7.160)	<0.001
South Africa	Referent	–
Female sex	−10.020 (7.647)	0.19
Marital status	2.215 (0.384)	<0.001
Living arrangements	−1.249 (0.666)	0.06
Socieconomic status		
1 (lowest)	Referent	–
2	−0.632 (0.416)	0.13
3	−0.833 (0.405)	0.04
4	−0.591 (0.397)	0.14
5 (highest)	−1.339 (0.406)	0.001
Family size	0.031 (0.044)	0.48

Age*sex*country interactions						
	Age*country		Sex*country		Age*sex*country	
	B (SE)	P	B (SE)	P	B (SE)	P
Bangladesh	0.584 (0.136)	<0.001	36.701 (14.663)	0.01	−0.100 (0.209)	0.63
Ghana	0.445 (0.156)	0.004	13.233 (12.621)	0.29	0.027 (0.169)	0.87
Indonesia	0.317 (0.105)	0.003	4.371 (8.032)	0.59	0.133 (0.044)	0.002
India	0.004 (0.134)	0.98	16.106 (11.195)	0.15	−0.056 (0.134)	0.68
Kenya	0.251 (0.150)	0.09	0.479 (14.608)	0.97	0.247 (0.220)	0.26
Tanzania	0.259 (0.114)	0.02	4.283 (9.014)	0.64	0.158 (0.083)	0.06
Vietnam	0.477 (0.116)	<0.001	5.332 (8.856)	0.55	0.130 (0.076)	0.09
South Africa	Referent	–	Referent	–	0.205 (0.123)	0.10

B, unstandardised coefficient; WHODAS, WHO Disability Assessment Schedule.

Sex, marital status and socioeconomic status were associated with disability scores; the relationship between age and disability across countries was more heterogeneous than seen with age-quality of life gradients; most sites bar India and Kenya showed significant age–disability score interactions. In contrast to quality of life, only Bangladesh showed a significant different in the relationship between disability and sex from that seen in South Africa, and only Indonesia showed a significant different from South Africa when the interaction of age, sex and country was considered.

Table 4 contains results of regression models both including and excluding anchoring vignettes. With the exception of India, we found little difference in the country-specific coefficients when the vignettes were included in the model.

**Table 4:** Impact of differences in health status vignette replies on disability scores across countries (n=8201)

Variable	Without adjusting for vignette differences		Adjusting for vignette differences	
	B (SE)	P	B (SE)	P
Country of residence				
Bangladesh	0.571 (0.067)	<0.001	0.442 (0.070)	<0.001
Indonesia	−0.440 (0.051)	<0.001	−0.551 (0.052)	<0.001
India	0.268 (0.067)	<0.001	−0.021 (0.071)	0.77
Kenya	−0.020 (0.140)	0.88	−0.151 (0.141)	0.28
Tanzania	−0.271 (0.079)	0.001	−0.363 (0.084)	0.003
Vietnam	Referent	–	Referent	–
Age	0.034 (0.002)	<0.001	0.030 (0.002)	<0.001
Female sex	0.277 (0.042)	<0.001	0.251 (0.041)	<0.001
Marital status	0.100 (0.051)	0.05	0.079 (0.051)	0.12
Living arrangements	−0.027 (0.092)	0.77	0.032 (0.092)	0.73
Socioeconomic status	−0.027 (0.014)	0.06	−0.022 (0.014)	0.12
Family size	0.002 (0.007)	0.81	0.003 (0.007)	0.63

No data for Ghana and South Africa.

B, unstandardised coefficient.

## Discussion

This analysis shows significant differences in patterns of both disability and quality of life in adults aged 50 years and over between eight LMICs. The overall pattern across sites was of increasing disability and worsening quality of life with age. Comparison of the relationship between these outcome variables and both age and sex showed differences between the pattern in the referent country (South Africa) and some other countries, most notably Bangladesh. The lack of effect of including anchoring vignette results in the analyses suggests that most differences between countries in reported disability were not explained by differences in how levels of impairment were perceived between countries.

Taken together, our analyses show that concepts of the impacts of ageing on quality of life and disability cannot be assumed to be the same across genders and across sociocultural and spatial contexts. This finding has important implications for governments in LMICs who are developing health and long-term care services to cope with increases in life expectancy and a growing population of older people. They also have important implications when considering the wider economy. For example, older people in LMICs contribute substantially to the formal and informal economies. Additionally, older women often provide childcare services that allow younger members of the family to work. These roles require older people to be active. Moreover, younger relatives (also usually women) who need to care for less able older people are often removed from the economy.^27^

Although this dataset did not include disease-specific diagnoses associated with disability and quality of life decline with increasing age, it is reasonable to assume in this over-50 age group that the observed changes are largely linked to chronic non-communicable conditions, as we discuss further below. Thus, these findings are also a highly relevant background to sustainable development goal (SDG) targets 3.4 and 3.8 (reducing non-communicable disease burdens and providing universal health coverage). Noting our findings that countries had very different patterns of age-related functional decline will be very important in understanding progress towards the SDGs.

We found that the differences in disability and quality of life between sites were not explained by differences in socioeconomic status, differences in age or sex structure or additionally (for quality of life) by variations in disability and selected indicators of health status. Previous analyses of this dataset have shown significant differences in overall health status between countries, with men tending to have better health status than women.^20^ These results therefore concur with the findings of the current analysis. It remains entirely possible though, as discussed further below, that differences between countries are mediated in part by differences in comorbid disease (a construct different from health status), which were not measured as part of the INDEPTH WHO-SAGE dataset.

Previous work shows a clear trend for increasing disability with age in both HICs and LMICs.^9^ A recent analysis of disability-free life expectancy in six LMICs (including three countries included in the current analysis) showed increasing levels of impairment in basic activities of daily living (ie, a related but distinct construct from that measured by WHODAS-II) with increasing age and with female sex; estimates of the prevalence of at least one impairment in basic activities of daily living (ADLs) varied from 13% in China to 54% in India.^18^ Rates of ADL impairment were higher for women in all six countries; rates of disability varied markedly between countries for those aged 50–54 years (range 5%–30% for men in this age group; range 8%–55% for women) but converged with increasing age (45%–80%)%) in the over-85 age group for both sexes. Few data are available using WHODAS-II and WHOQoL in HICs, but median WHODAS-II disability scores from an Australian study^2^ were much lower (better) at any given age than those observed for any site in our analysis. Data from a validation study^24^ of the eight-item WHOQoL tool used in the current study in six middle-income countries to HICs (Israel, Spain, Australia, Brazil, USA and Russia) showed a range of mean scores in non-depressed participants across countries: from 2.98/5 (equivalent to 60/100) in Russia to 3.63/5 (equivalent to 73/100) in Israel. This analysis did not attempt to examine differences with age or sex in a population-representative sample however.

What other factors might explain the differences between sites in our analysis? It is possible that differences in the burden of disability between countries reflect differences in the biological age (as opposed to the chronological age) of participants; in other words, disease burden from both communicable and non-communicable disease may weigh more heavily on some populations at a given age. However, countries with low disability rates (eg, Indonesia and India) in our analysis had life expectancies at age 60 years that were no better (and in some cases worse) than countries with more disability.^28^ The contrast between Bangladesh and its neighbours is particularly noteworthy, and we cannot explain the magnitude of this difference with the existing data. A number of other factors, unmeasured in this analysis, may contribute to the differences seen—particularly around physical activity, obesity, smoking, alcohol use and burden of particular non-communicable diseases (NCDs) likely to contribute to disability (eg, stroke, chronic obstructive pulmonary disease and heart failure). Environmental factors across the lifecourse, including housing, pollution, and working conditions, may also have an impact, and the ability of healthcare systems to address or mitigate the effects of these factors is also likely to be important. Differences in patterning of these risk factors across the life course may also partly explain the differences in disability scores between men and women, as might differential dropout; those with the most severe disabilities are likely to either die or be unable to participate in surveys. Several other studies have noted higher disability rates at all ages among women,^4 18 29 30^ and a number of possible explanations have been advanced for this. Women have a lower peak muscle mass in early adulthood and may be more likely to suffer from impaired physical function in later life as they can afford to lose less muscle strength before a threshold of functional impairment is reached.^31^ Another possible explanation is that women are able to tolerate a higher burden of frailty before system failure (ie, death) supervenes.^32^ Differential dropout caused by shorter life expectancy in men in many countries may then also contribute; the surviving men at any given age are likely to be fitter and less disabled.

Although illness and disability are important factors affecting overall quality of life, other factors (eg, income, social connectedness and mood) are equally, if not more, important in determining overall quality of life or life satisfaction.^33–35^ It is not clear from this analysis which of these factors might explain the findings, but possible explanations include the expectations, roles and contributions of older people in different societies, how disability and dependency modify societal roles for older people in different cultures, how older people are cared for by both formal and informal health and care systems, the extent of older people’s integration into family and social networks and the effect of urbanisation and technology on how older people are able to live and age successfully. Another possible explanation as to why patterns of quality of life differ between countries may be that the construct of quality of life is interpreted differently in different countries or is interpreted differently by men and women between countries.

### Strengths and limitations

Our work has several strengths. Our large sample is drawn from eight different countries representing a diversity of culture, geography and economic development. The same questionnaires were applied at all sites, using validated tools, the populations at each site were part of an HDSS, and the selected sample was representative of the surveyed population of older people at each site.

A number of limitations also require discussion. The cross-sectional nature of the data does not allow causal inference to be drawn, and the covariates that we chose to include were based on a priori hypotheses, driven by previous research in other populations. We are not able to tell whether these covariates were causally associated with either future disability or quality of life in this cohort. The number of covariates that we were able to access was limited by the nature of the data present in the INDEPTH WHO-SAGE dataset. Key measures that were missing and would be helpful to include in future analyses include comorbid disease, cardiometabolic risk factors, measures of frailty, more sophisticated measures of social networks, including caregiving by family members and others, and more in-depth analysis of the components of socioeconomic status. Poor mental health is known to be associated with worse quality of life and function, and this is a key issue for future surveys to explore in more depth. Although the surveys that comprised this dataset were broadly representative of the populations sampled, it is likely that those who were most disabled may have been less likely to participate. Similarly, although institutional care is uncommon in the populations sampled, the sampling strategy will have failed to include those in institutional care or hospital, again possibly underestimating levels of disability although the impact of institutionalisation is likely to be small. In addition, data were drawn from HDSS sites, which study a specific (resource-limited) population within a country in detail and are not necessarily representative of a country population as a whole. The data used in this analysis are now 10 years old, and rapid changes in health and socioeconomic circumstances in the included sites are likely to have affected both disability and quality of life in the last 10 years. Nevertheless, our results are based on the most recent data we could access and act as a baseline with which to compare future measurements of disability and quality of life comparisons between these sites.

Although the use of anchoring vignettes is an attractive concept to allow adjustment for differences in perception of disability between individuals and cultures, the reliability of the information provided by such vignettes has been questioned, as their performance does not necessarily meet stringent thresholds for psychometric performance. Nevertheless, use of such vignettes may still provide useful information on the way that individuals across countries vary in how they order the severity of disability even if their ability to adjust for absolute values of disability scores is limited.^25^ Even with the use of anchoring vignettes, between-country differences in how the constructs of disability and quality of life are interpreted may explain some of the between-country differences. We were unable to compare the effect of vignette responses across all countries, as vignettes were used in only six of the eight sites. Newer tools for measuring disability (eg, WHODAS 2.0), developed using Item Response Theory,^36^ may also circumvent some of the variability in interpretation of questions across countries. Large-scale cross-sectional analyses are limited in their ability to explore how the construct of disability is mediated by the interplay of biology and culture, and in-depth, focused qualitative studies are likely to be a more fruitful way to explore these issues further.

## Conclusions

Rapid demographic and socioeconomic transitions are occurring in all of the countries included in this analysis: several countries (eg, Ghana, Bangladesh and Kenya) that were classed as low-income countries at the time of data collection are now classed as LMICs; South Africa has moved from the middle to upper-middle income bracket; and life expectancy is rising rapidly in many countries. A key next step is therefore to obtain and analyse longitudinal data, and planned future waves of INDEPTH WHO-SAGE should hopefully allow collection of these data. Such analyses will be able to test how secular trends are changing the distribution of disability and quality of life between countries and will also be able to map how disability and quality of life change for older individuals over time. Such analyses, together with collection of a broader range of potential explanatory factors, will shed further light on the determinants of disability and quality of life between countries. This in turn will create opportunities to shape interventions and policy to minimise disability and maximise quality of life for older people as the numbers of older people continue to rise.

## References

[1] GBD 2015 Mortality and Causes of Death Collaborators. Global, regional, and national life expectancy, all-cause mortality, and cause-specific mortality for 249 causes of death, 1980-2015: a systematic analysis for the global burden of disease study 2015. Lancet 2016;388:1459–544. 10.1016/S0140-6736(16)31012-1 27733281 PMC5388903

[2] Andrews G, Kemp A, Sunderland M, et al Normative data for the 12 item WHO disability assessment schedule 2.0. PLoS One 2009;4:e8343 10.1371/journal.pone.0008343 20020047 PMC2791224

[3] Chatterji S, Byles J, Cutler D, et al Health, functioning, and disability in older adults-present status and future implications. Lancet 2015;385:563–75. 10.1016/S0140-6736(14)61462-8 25468158 PMC4882096

[4] Van Oyen H, Nusselder W, Jagger C, et al Gender differences in healthy life years within the EU: an exploration of the “health-survival” paradox. Int J Public Health 2013;58:143–55. 10.1007/s00038-012-0361-1 22618297 PMC3557379

[5] Roberts H, Khee TS, Philip I Setting priorities for measures of performance for geriatric medical services. Age Ageing 1994;23:154–7. 10.1093/ageing/23.2.154 8023726

[6] Hirve S, Juvekar S, Sambhudas S, et al Does self-rated health predict death in adults aged 50 years and above in India? Evidence from a rural population under health and demographic surveillance. Int J Epidemiol 2012;41:1719–27. 10.1093/ije/dys163 23175517 PMC3621387

[7] Gómez-Olivé FX, Thorogood M, Bocquier P, et al Social conditions and disability related to the mortality of older people in rural South Africa. Int J Epidemiol 2014;43:1531–41. 10.1093/ije/dyu093 24836326 PMC4190514

[8] Wouterse B, Huisman M, Meijboom BR, et al The effect of trends in health and longevity on health services use by older adults. BMC Health Serv Res 2015;15:574 10.1186/s12913-015-1239-8 26704342 PMC4690430

[9] World Health Organisation. Global strategy and action plan on ageing and health. 2016 http://www.who.int/ageing/global-strategy/en/ (accessed 2 Aug 2017).

[10] Xavier Gómez-Olivé F, Thorogood M, Clark BD, et al Assessing health and well-being among older people in rural South Africa. Glob Health Action 2010;3(Suppl 2):23–35. 10.3402/gha.v3i0.2126 PMC2957314 20963188

[11] Mwanyangala MA, Mayombana C, Urassa H, et al Health status and quality of life among older adults in rural Tanzania. Glob Health Action 2010;3(Suppl 2):36–44. 10.3402/gha.v3i0.2142 PMC2958089 20975983

[12] Kyobutungi C, Egondi T, Ezeh A The health and well-being of older people in Nairobi’s slums. Glob Health Action 2010;3(Suppl 2):45–53. 10.3402/gha.v3i0.2138 PMC2957141 20959873

[13] Debpuur C, Welaga P, Wak G, et al Self-reported health and functional limitations among older people in the Kassena-Nankana District, Ghana. Glob Health Action 2010;3(Suppl 2):54–63. 10.3402/gha.v3i0.2151 PMC2957305 20963186

[14] Van Minh H, Byass P, Chuc NT, et al Patterns of health status and quality of life among older people in rural Viet Nam. Glob Health Action 2010;3(Suppl 2):64–9. 10.3402/gha.v3i0.2124 PMC2957159 20959877

[15] Razzaque A, Nahar L, Akter Khanam M, et al Socio-demographic differentials of adult health indicators in Matlab, Bangladesh: self-rated health, health state, quality of life and disability level. Glob Health Action 2010;3(Suppl 2):70–7. 10.3402/gha.v3i0.4618 PMC2958199 20975960

[16] Ng N, Hakimi M, Byass P, et al Health and quality of life among older rural people in Purworejo District, Indonesia. Glob Health Action 2010;3:2125–87. 10.3402/gha.v3i0.2125 PMC2957148 20959875

[17] Hirve S, Juvekar S, Lele P, et al Social gradients in self-reported health and well-being among adults aged 50 and over in Pune District, India. Glob Health Action 2010;3:2128–95. 10.3402/gha.v3i0.2128 PMC2958441 20975980

[18] Santosa A, Schröders J, Vaezghasemi M, et al Inequality in disability-free life expectancies among older men and women in six countries with developing economies. J Epidemiol Community Health 2016;70:855–61. 10.1136/jech-2015-206640 26994068 PMC5013163

[19] Kowal P, Kahn K, Ng N, et al Ageing and adult health status in eight lower-income countries: the INDEPTH WHO-SAGE collaboration. Glob Health Action 2010;3(Suppl 2):11–22. 10.3402/gha.v3i0.5302 PMC2957285 20959878

[20] Ng N, Kowal P, Kahn K, et al Health inequalities among older men and women in Africa and Asia: evidence from eight Health and Demographic Surveillance System sites in the INDEPTH WHO-SAGE Study. Glob Health Action 2010;3:5420–107. 10.3402/gha.v3i0.5420 PMC2958198 20967141

[21] Garin O, Ayuso-Mateos JL, Almansa J, et al Validation of the "World Health Organization Disability Assessment Schedule, WHODAS-2" in patients with chronic diseases. Health Qual Life Outcomes 2010;8:51 10.1186/1477-7525-8-51 20482853 PMC2893517

[22] Schmidt S, Mühlan H, Power M The EUROHIS-QOL 8-item index: psychometric results of a cross-cultural field study. Eur J Public Health 2006;16:420–8. 10.1093/eurpub/cki155 16141303

[23] The WHOQoL group. Development of the world health organization WHOQOL-BREF quality of life assessment. Psychol Med 1998;28:551–8. 9626712 10.1017/s0033291798006667

[24] da Rocha NS, Power MJ, Bushnell DM, et al The EUROHIS-QOL 8-item index: comparative psychometric properties to its parent WHOQOL-BREF. Value Health 2012;15:449–57. 10.1016/j.jval.2011.11.035 22583455

[25] Hirve S, Gómez-Olivé X, Oti S, et al Use of anchoring vignettes to evaluate health reporting behavior amongst adults aged 50 years and above in Africa and Asia - testing assumptions. Glob Health Action 2013;6:21064 10.3402/gha.v6i0.21064 24011254 PMC3765649

[26] World Bank Data Bank. Explore create share: development data. 2017 http://databank.worldbank.org/data/home.aspx.

[27] Langer A, Meleis A, Knaul FM, et al Women and Health: the key for sustainable development. Lancet 2015;386:1165–210. 10.1016/S0140-6736(15)60497-4 26051370

[28] World Health Organisation. Global health observatory data repository. http://apps.who.int/gho/data/node.main.688 (accessed 17 Apr 2017).

[29] Hosseinpoor AR, Williams JS, Jann B, et al Social determinants of sex differences in disability among older adults: a multi-country decomposition analysis using the world health survey. Int J Equity Health 2012;11:52 10.1186/1475-9276-11-52 22958712 PMC3463479

[30] Smith MP, Olatunde O, White C Disability‐free life expectancy: comparison of sources and small area estimates in England, 2006–08. Health Stat Q 2011;50:40–78. 10.1057/hsq.2011.8 21647088

[31] Dodds RM, Syddall HE, Cooper R, et al Global variation in grip strength: a systematic review and meta-analysis of normative data. Age Ageing 2016;45:209–16. 10.1093/ageing/afv192 26790455 PMC4776623

[32] Hubbard RE, Rockwood K Frailty in older women. Maturitas 2011;69:203–7. 10.1016/j.maturitas.2011.04.006 21570783

[33] Raggi A, Corso B, Minicuci N, et al Determinants of quality of life in ageing populations: results from a cross-sectional study in Finland, Poland and Spain. PLoS One 2016;11:e0159293 10.1371/journal.pone.0159293 27434374 PMC4951007

[34] Conde-Sala JL, Portellano-Ortiz C, Calvó-Perxas L, et al Quality of life in people aged 65+ in Europe: associated factors and models of social welfare-analysis of data from the SHARE project (Wave 5). Qual Life Res 2017;26:1059–70. 10.1007/s11136-016-1436-x 27766517

[35] Lamu AN, Olsen JA The relative importance of health, income and social relations for subjective well-being: an integrative analysis. Soc Sci Med 2016;152:176–85. 10.1016/j.socscimed.2016.01.046 26854627

[36] Reeve BB, Hays RD, Chang C-H, et al Applying item response theory to enhance health outcomes assessment. Quality of Life Research 2007;16:1–3. 10.1007/s11136-007-9220-6 17033892

