# Supplementary material for: Variations in disability and quality of life with age and sex between eight lower income and middle-income countries: data from the INDEPTH WHO-SAGE collaboration
Source: BMJ Glob Health. 2017 Dec 20;2(4):e000508. doi: 10.1136/bmjgh-2017-000508 (PMC5759706; doi:10.1136/bmjgh-2017-000508)
Supplement: Supplementary file 1 [file bmjgh-2017-000508supp001.pdf]

Supplementary Table 1. Associations with WHOQoL scores – regression models run for individual countries

		Bangladesh	Ghana	Indonesia	India	Kenya	Tanzania	Vietnam	South Africa
Variable		B (95% CI)	B (95% CI)	B (95% CI)	B (95% CI)	B (95% CI)	B (95% CI)	B (95% CI)	B (95% CI)
Intercept		**77.89<br>(75.87, 79.91)**	**78.33<br>(76.37, 80.29)**	**75.05<br>(74.13, 75.97)**	**78.46<br>(77.10, 79.83)**	**79.23<br>(76.85, 81.61)**	**74.10<br>(72.03, 76.18)**	**68.25<br>(66.95, 69.55)**	**71.71<br>(69.27, 74.16)**
Age (per year)		-0.03<br>(-0.06, 0.00)	-0.03<br>(-0.06, 0.00)	0.01<br>(0.00, 0.03)	-0.02<br>(-0.04, 0.00)	-0.02<br>(-0.06, 0.02)	**-0.04<br>(-0.07, -0.01)**	**0.07<br>(0.05, 0.09)**	0.03<br>(-0.01, 0.06)
Female sex		**3.65<br>(0.93, 6.37)**	-2.30<br>(-4.77, 0.16)	**-1.38<br>(-2.53, -0.22)**	-0.09<br>(-1.96, 1.78)	0.17<br>(-3.15, 3.49)	**-4.72<br>(-7.18, -2.27)**	**-3.08<br>(-4.58, -1.57)**	0.08<br>(-2.58, 2.74)
Education	None	**-0.82<br>(-1.42, -0.21)**	-*	**-0.59<br>(-0.92, -0.26)**	0.14<br>(-0.49, 0.77)	**-1.25<br>(-2.07, -0.42)**	-0.07<br>(-1.01, 0.88)	**-1.53<br>(-2.05, -1.02)**	-0.29<br>(-0.82, 0.26)
	<6 years	-0.34<br>(-0.96, 0.28)	**-1.07<br>(-1.74, -0.39)**	-0.17<br>(-0.45, 0.10)	**-0.47<br>(-0.80, -0.13)**	-0.65<br>(-1.33, 0.04)	-0.25<br>(-1.16, 0.65)	**-0.97<br>(-1.27, -0.68)**	-0.37<br>(-0.98, 0.24)
	>=6 years	Referent	Referent	Referent	Referent	Referent	Referent	Referent	Referent
Marital status=2		**-6.36<br>(-6.93, -5.79)**	-0.35<br>(-0.76, 0.07)	-0.16<br>(-0.39, 0.07)	-0.33<br>(-0.70, 0.04)	**-0.87<br>(-1.54, -0.20)**	-0.18<br>(-0.62, 0.25)	**-0.68<br>(-1.00, -0.37)**	**-0.63<br>(-1.06, -0.21)**
Living arrangements=1		0.99<br>(-0.23, 2.20)	-0.44<br>(-1.30, 0.43)	-0.05<br>(-0.44, 0.34)	0.55<br>(-0.41, 1.52)	-0.56<br>(-1.22, 0.11)	-0.06<br>(-1.33, 1.22)	0.55<br>(-0.01, 1.11)	0.66<br>(-0.22, 1.53)
Socioeconomic status quintiles	1 (lowest)	Referent	Referent	Referent	Referent	Referent	Referent	Referent	Referent
	2	**1.02<br>(0.35, 1.69)**	0.17<br>(-0.31, 0.64)	**0.63<br>(0.36, 0.90)**	0.45<br>(-0.07, 0.97)	**-1.21<br>(-2.00, -0.43)**	-0.04<br>(-0.62, 0.54)	**1.57<br>(1.15, 1.99)**	0.50<br>(-0.13, 1.13)
	3	**2.29<br>(1.63, 2.96)**	**0.74<br>(0.26, 1.23)**	**1.45<br>(1.18, 1.72)**	0.46<br>(-0.02, 0.94)	**-0.72<br>(-1.43, -0.01)**	-0.42<br>(-0.99, 0.15)	**2.30<br>(1.87, 2.73)**	**1.46<br>(0.82, 2.09)**
	4	**2.95<br>(2.31, 3.59)**	**1.69<br>(1.18, 2.21)**	**1.69<br>(1.41, 1.97)**	**0.65<br>(0.15, 1.14)**	-0.04<br>(-0.75, 0.67)	-0.42<br>(-0.95, 0.10)	**2.53<br>(2.10, 2.95)**	**1.31<br>(0.67, 1.95)**
	5 (highest)	**4.35<br>(3.70, 4.99)**	**2.78<br>(1.99, 3.56)**	**2.63<br>(2.32, 2.93)**	**0.78<br>(0.30, 1.26)**	-0.19<br>(-0.91, 0.54)	-*	**4.08<br>(3.65, 4.51)**	**2.23<br>(1.61, 2.86)**
Family size (per person)		-0.01<br>(-0.09, 0.07)	**-0.05<br>(-0.10, -0.00)**	**-0.07<br>(-0.12, -0.01)**	-0.01<br>(-0.05, 0.03)	-0.09<br>(-0.18, 0.00)	-0.02<br>(-0.05, 0.01)	**-0.20<br>(-0.27, -0.13)**	**-0.06<br>(-0.11, -0.01)**
WHODAS-II score		**-0.17<br>(-0.18, -0.16)**	**-0.26<br>(-0.27, -0.25)**	**-0.16<br>(-0.17, -0.16)**	**-0.11<br>(-0.12, -0.10)**	**-0.22<br>(-0.23, -0.20)**	**-0.16<br>(-0.17, -0.15)**	**-0.19<br>(-0.19, -0.18)**	**-0.20<br>(-0.21, -0.19)**
Age*sex		**-0.05<br>(-0.10, -0.01)**	0.03<br>(-0.01, 0.07)	**0.03<br>(0.01, 0.04)**	0.01<br>(-0.03, 0.04)	-0.01<br>(-0.07, 0.05)	**0.08<br>(0.04, 0.12)**	**0.04<br>(0.02, 0.07)**	0.01<br>(-0.03, 0.05)

B: Unstandardised coefficient

Bold: p<0.05

*no individuals in this category

Supplementary Table 2. Associations with WHODAS-II scores – regression models run for individual countries

		Bangladesh	Ghana	Indonesia	India	Kenya	Tanzania	Vietnam	South Africa
Variable		B (95% CI)	B (95% CI)	B (95% CI)	B (95% CI)	B (95% CI)	B (95% CI)	B (95% CI)	B (95% CI)
Intercept		**-26.33**<br>**(-35.06, -17.59)**	**-20.52**<br>**(-29.80, -11.25)**	**-24.27**<br>**(-28.70, -19.85)**	6.34<br>(-0.74, 13.42)	**-18.01**<br>**(-31.95, -4.07)**	**-18.71**<br>**(-26.99, -10.44)**	**-23.54**<br>**(-30.48, -16.60)**	3.75<br>(-7.47, 14.96)
Age (per year)		**0.81**<br>**(0.69, 0.94)**	**0.67**<br>**(0.51, 0.81)**	**0.54**<br>**(0.47, 0.61**	**0.23**<br>**(0.13, 0.34)**	**0.47**<br>**(0.23, 0.71)**	**0.48**<br>**(0.34, 0.61)**	**0.69**<br>**(0.58, 0.79)**	**0.22**<br>**(0.05, 0.38)**
Female sex		**24.80**<br>**(7.05, 42.56)**	4.21<br>(-8.45, 16.86)	-5.25<br>(-11.34, 0.84)	5.32<br>(-4.77, 15.41)	-8.85<br>(-35.46, 17.77)	-5.22<br>(-17.51, 7.08)	-5.40<br>(-13.73, 2.92)	-10.14<br>(-22.73, 2.44)
Marital status=2		0.67<br>(-3.41, 4.75)	**3.06**<br>**(0.98, 5.15)**	**2.43**<br>**(0.93, 3.92)**	0.62<br>(-1.37, 2.61)	3.89<br>(-0.11, 7.88)	1.69<br>(-0.42, 3.80)	1.67<br>(-0.44, 3.78)	1.98<br>(-0.22, 4.18)
Living arrangements=1		-6.25<br>(-15.34, 2.85)	-1.37<br>(-5.81, 3.07)	-0.89<br>(-3.45, 1.67)	-2.39<br>(-7.46, 2.68)	0.24<br>(-3.20, 3.67)	-3.86<br>(-8.56, 0.83)	-1.58<br>(-5.46, 2.29)	-2.15<br>(-6.60, 2.31)
Socioeconomic status quintiles	1 (lowest)	Referent	Referent	Referent	Referent	Referent	Referent	Referent	Referent
	2	-1.36<br>(-4.81, 2.10)	0.96<br>(-1.29, 3.21)	-0.83<br>(-2.30, 0.64)	1.95<br>(-0.80, 4.70)	-1.41<br>(-5.65, 2.82)	-1.28<br>(-3.52, 0.96)	-1.31<br>(-4.03, 1.41)	0.33<br>(-2.85, 3.52)
	3	-1.60<br>(-4.98, 1.80)	1.77<br>(-0.59, 4.13)	-1.30<br>(-2.72, 0.12)	0.74<br>(-1.79, 3.26)	-1.03<br>(-4.64, 2.57)	-0.30<br>(-2.72, 2.11)	-2.23<br>(-4.91, 0.45)	0.71<br>(-2.48, 3.90)
	4	-2.18<br>(-5.37, 1.02)	0.61<br>(-1.83, 3.05)	-1.35<br>(-2.75, 0.04)	0.09<br>(-2.46, 2.64)	-0.39<br>(-4.14, 3.37)	1.09<br>(-1.18, 3.36)	-2.37<br>(-5.01, 0.27)	1.64<br>(-1.61, 4.89)
	5 (highest)	**-3.95**<br>**(-7.04, -0.86)**	0.08<br>(-3.68, 3.85)	-1.34<br>(-2.72, 0.05)	-0.48<br>(-2.95, 1.98)	-0.46<br>(-4.07, 3.16)	-	**-3.69**<br>**(-6.30, -1.08)**	-0.13<br>(-3.15, 2.90)
Family size (per person)		0.00<br>(-0.40, 0.41)	0.03<br>(-0.19, 0.26)	0.20<br>(-0.08, 0.47)	-0.02<br>(-0.23, 0.19)	0.33<br>(-0.17, 0.83)	0.03<br>(-0.13, 0.19)	-0.16<br>(-0.53, 0.21)	-0.06<br>(-0.32, 0.20)
Age*sex		-0.06<br>(-0.36, 0.25)	0.00<br>(-0.21, 0.22)	**0.13**<br>**(0.02, 0.23)**	-0.04<br>(-0.20, 0.13)	0.23<br>(-0.25, 0.70)	0.15<br>(-0.06, 0.37)	0.14<br>(0.00, 0.29)	**0.21**<br>**(0.01, 0.41)**

B: Unstandardised coefficient

Bold: p<0.05

Supplementary Table 3. Alternative WHOQOL analyses using individual health status components

Variable		B (SE)	P
Age (per year)		0.025 (0.017)	0.14
Country of residence			
- Bangladesh		6.680 (1.374)	<0.001
- Ghana		9.383 (1.412)	<0.001
- Indonesia		4.978 (1.201)	<0.001
- India		5.167 (1.350)	<0.001
- Kenya		5.262 91.604)	0.001
- Tanzania		2.819 (1.349)	0.04
- Vietnam		0.661 (1.258)	0.60
- South Africa		Referent	-
Female sex		1.928 (1.263)	0.13
Education	None	-1.069 (0.098)	<0.001
	<6 years	-0.592 (0.079)	<0.001
	>=6 years	Referent	-
Marital status		-0.953 (0.069)	<0.001
Living arrangements		0.470 (0.123)	<0.001
Socieconomic status	1 (lowest)	Referent	-
	2	0.430 (0.086)	<0.001
	3	0.942 (0.085)	<0.001
	4	1.192 (0.084)	<0.001
	5 (highest)	2.142 (0.092)	<0.001
Family size		-0.039 (0.009)	<0.001
Depressive symptoms		-0.674 (0.042)	<0.001
Anxiety symptoms		-0.804 (0.040)	<0.001
Bodily pain		-0.497 (0.044)	<0.001
Discomfort		-0.891 (0.046)	<0.001
Problems concentrating		-0.420 (0.036)	<0.001
Problems learning a new task		-0.347 (0.032)	<0.001
Problems far vision		-0.443 (0.033)	<0.001
Problems near vision		-0.412 (0.034)	<0.001
Age, sex & country interactions			

	Age*country		Sex*country		Age*sex*country	
	B (SE)	p	B (SE)	p	B (SE)	p
- Bangladesh	-0.117 (0.021)	<0.001	6.401 (1.751)	<0.001	-0.166 (0.019)	<0.001
- Ghana	-0.158 (0.022)	<0.001	-5.033 (1.729)	0.004	0.041 (0.019)	0.03
- Indonesia	-0.029 (0.019)	0.11	-1.890 (1.434)	0.19	0.007 (0.011)	0.51
- India	-0.014 (0.021)	0.50	-1.525 (1.704)	0.37	0.005 (0.018)	0.79
- Kenya	-0.063 (0.026)	0.02	-0.233 (2.182)	0.92	-0.038 (0.030)	0.20
- Tanzania	-0.072 (0.021)	0.001	-5.340 (1.644)	0.001	0.063 (0.017)	<0.001
- Vietnam	-0.036 (0.019)	0.07	-3.040 (1.478)	0.04	0.018 (0.012)	0.14
- South Africa	Referent	-	Referent	-	-0.016 (0.020)	0.42

B: Unstandardised coefficient. SE: Standard error

N=43622. Data on one or more health status measures missing for 313 individuals

Supplementary Table 4. Alternative WHOQOL analyses using integrated health status measure

Variable		B (SE)	P
Age (per year)		0.019 (0.017)	0.27
Country of residence			
- Bangladesh		7.199 (1.412)	<0.001
- Ghana		9.535 (1.455)	<0.001
- Indonesia		2.707 (1.235)	0.03
- India		4.839 (1.390)	0.001
- Kenya		3.680 (1.655)	0.03
- Tanzania		1.702 (1.387)	0.22
- Vietnam		-0.547 (1.295)	0.67
- South Africa		Referent	-
Female sex		1.754 (1.296)	0.18
Education	None	-1.132 (0.101)	<0.001
	<6 years	-0.578 (0.082)	<0.001
	>=6 years	Referent	-
Marital status		-0.929 (0.071)	<0.001
Living arrangements		0.325 (0.127)	0.01
Socieconomic status	1 (lowest)	Referent	-
	2	0.461 (0.089)	<0.001
	3	0.947 (0.088)	<0.001
	4	1.235 (0.087)	<0.001
	5 (highest)	2.225 (0.095)	<0.001
Family size		-0.036 (0.009)	<0.001
Health status score		0.263 (0.003)	<0.001

*Age*sex*country interactions*

	Age*country		Sex*country		Age*sex*country	
	B (SE)	p	B (SE)	p	B (SE)	p
- Bangladesh	-0.130 (0.022)	<0.001	4.982 (1.799)	0.006	-0.166 (0.020)	<0.001
- Ghana	-0.150 (0.023)	<0.001	-4.149 (1.782)	0.02	0.027 (0.019)	0.16
- Indonesia	0.009 (0.019)	0.65	-1.518 (1.473)	0.30	0.007 (0.011)	0.53
- India	-0.002 (0.022)	0.92	-1.046 (1.756)	0.55	0.001 (0.019)	0.95
- Kenya	-0.038 (0.027)	0.16	1.079 (2.253)	0.63	-0.057 (0.031)	0.07

- Tanzania	-0.055 (0.022)	0.01	-5.483 (1.688)	0.001	0.072 (0.017)	<0.001
- Vietnam	-0.011 (0.020)	0.60	-2.122 (1.520)	0.16	0.003 (0.012)	0.80
- South Africa	Referent	-	Referent	-	-0.016 (0.020)	0.41

B: Unstandardised coefficient. SE: Standard error
